# Fit for Discharge?: A First-Cycle Audit Investigating the Documentation of Delirium in Medical Discharge Letters

**DOI:** 10.1192/bjo.2025.10566

**Published:** 2025-06-20

**Authors:** Rachael Curry

**Affiliations:** NHS South Tees, Northallerton, United Kingdom

## Abstract

**Aims:** Delirium is an acute confusional state, characterised by impacting the affected individual’s cognition and consciousness level to varying degrees over the course of an episode. Certain patient populations are more vulnerable to its development, of which the elderly are at particular risk. There are many factors that both predispose to and perpetuate a delirium, including having experienced it before. Not only does experiencing delirium once increase an individual’s risk of experiencing it again, but it also increases the risk of developing progressive, terminal cognitive conditions such as dementia. Strong associations between delirium and increase in overall morbidity have also been confirmed in recent literature.

Despite this, recognition and documentation of inpatient delirium still has room to improve. There is ongoing investigation into the recognition, documentation and appropriate investigation/monitoring of delirium at my clinical base, Friarage Hospital (Northallerton), which is reflected both regionally and nationally. Even when delirium is diagnosed, monitored and investigated with the input of the psychiatry team, the documentation in the discharge letters from the ward often does not reflect this part of the patient experience: compromising vital opportunity for pattern recognition and early intervention.

Aims were: To determine the consistency in documentation of delirium in elderly patients who have had psychiatric involvement whilst admitted.

To advocate for awareness of inpatient delirium as a predictor for morbidity, specifically:

1. Further episodes of delirium on subsequent admissions.

2. Development of chronic cognitive conditions, such as dementia.

**Methods:** Retrospective analysis of all patients discharged from the medical wards at FHN in November 2024, with the following inclusion criteria:

Over the age of 65 years old.

Admitted for an organic illness to medical ward in Friarage Hospital, Northallerton.

Referred to psychiatric liaison services during inpatient stay.

Discharge letter completed by ward upon completion of inpatient stay.

The appropriate patient population was pulled from Liaison Team records of patient reviews. This was a total of 60 patients in the month of November 2024.

The documentation of the respective patients’ inpatient stays was reviewed, using Miya and Patientrack for any mention of delirium or its varying presentations (confusion/agitation/aggression). Their discharge letters were then reviewed for any corresponding acknowledgement of this condition.

**Results:** 38 patients fit the inclusion criteria (above) in November 2024. When reviewing the Miya profiles of the selected patients, it was documented that 22 of these 38 (58%) experienced delirium during their inpatient stay.

Of those who experienced delirium, 13 (59%) had documentation of this in their final discharge letters. The remaining 9 patients (41%) had no mention of delirium OR its various presentations (confusion/agitation/aggression) in their final discharge letters.

The population was taken from all inpatient medical wards in the Friarage Hospital, Northallerton. This included the CDU (Clinical Decisions Unit) and the UTC (Urgent Treatment Centre), as well as the following wards: Romanby, Ainderby, Rutson.

In terms of patient location, the following was noted: 8 of the 22 patients with documentation of delirium were discharged from Romanby ward, 6 from Ainderby, 1 from Rutson, and 7 from CDU/UTC. The breakdown in documentation consistency was as follows:

Out of the 8 discharged from Romanby, 2 (25%) had appropriate documentation and 6 (75%) did not have documentation in their discharge letters.

Out of the 6 discharged from Ainderby, 5 (83%) had appropriate documentation and 1 (17%) did not have documentation in their discharge letters.

The single patient discharged from Rutson did not have documentation in their discharge letter.

Out of the 7 discharged from CDU and UTC, 6 (86%) had appropriate documentation and 1 (14%) did not have documentation in their discharge letters.

**Conclusion:** Understand that this is the patient population wherein the diagnosis of delirium is taken for granted, as it was recognised and investigated, with referrals made to psychiatry for management. There is likely a larger patient population who have experienced delirium as an inpatient: those who may not have been referred for psychiatric assessment or even been noted to have delirium.

Having aimed to assess the consistency of the existing documentation, there is hope that – by working backwards – can move to improve documentation of known cases initially and ultimately increase awareness and recognition of the condition in those who otherwise would not have been assessed. There is exciting potential in broadening the scope of this audit to cover all medical admissions.

Actions prior to re-audit (projected to be March 2025):

Education for ward staff in importance of including delirium/dementia in discharge summaries:

Teaching session for Foundation Year doctors in regards to documentation in discharges.

Informational posters on wards from 08/01/25.

Encourage documentation of previous episodes of delirium upon clerking (as other comorbidities are documented in ‘Problems’ section), even if patient doesn’t immediately present as confused.

In the small population audited, results demonstrated that only a slim majority (59%) of patients who required psychiatric input for their delirium had this documented in their medical discharge letters.

We hope to re-audit in March 2025 (as a retrospective of discharges in February 2025), giving minimum of 1 month to assess for change to practice.

